# Blood lactate levels and/or norepinephrine requirements for risk stratification in sepsis

**DOI:** 10.1186/cc14706

**Published:** 2015-09-28

**Authors:** Diego O Cortés, Arthur Cezar M Xavier, Bruno R de Almeida, Érica C de Vieira, Jacques Creteur, Jean-Louis Vincent, João Cláudio Lyra, Sylmara Zandona

**Affiliations:** 1 Department of Intensive Care, Hôpital Erasme, Anderlecht, Brussels, Belgium

## Introduction

Recent large multicenter studies on early resuscitation protocols for sepsis in the emergency room (ER) have shown a mortality rate of 19 % in the control groups [1,2]. These results suggest that the strategies used to include patients in these studies (high lactate or use of norepinephrine) did not identify a population at high risk of mortality. We explored the prognostic values of these criteria in an ICU population.

## Methods

All admissions to our department of intensive care in 2013 were retrospectively screened to identify patients who had an initial elevated lactate (≥2 mEq/l) *or *needed norepinephrine infusion (group OR) vs. those who had an initial elevated lactate *and *needed norepinephrine infusion (group AND) during the first 24 hours. We then classified the groups by the presence of sepsis at admission or not. The analysis was repeated using a lactate threshold of ≥4 mEq/l. We collected relevant demographic and clinical data including the type of admission, data needed to calculate the sequential organ failure assessment (SOFA) score, and ICU mortality. All values are presented as proportions or median values (percentiles 25-75).

## Results

**Figure 1:**
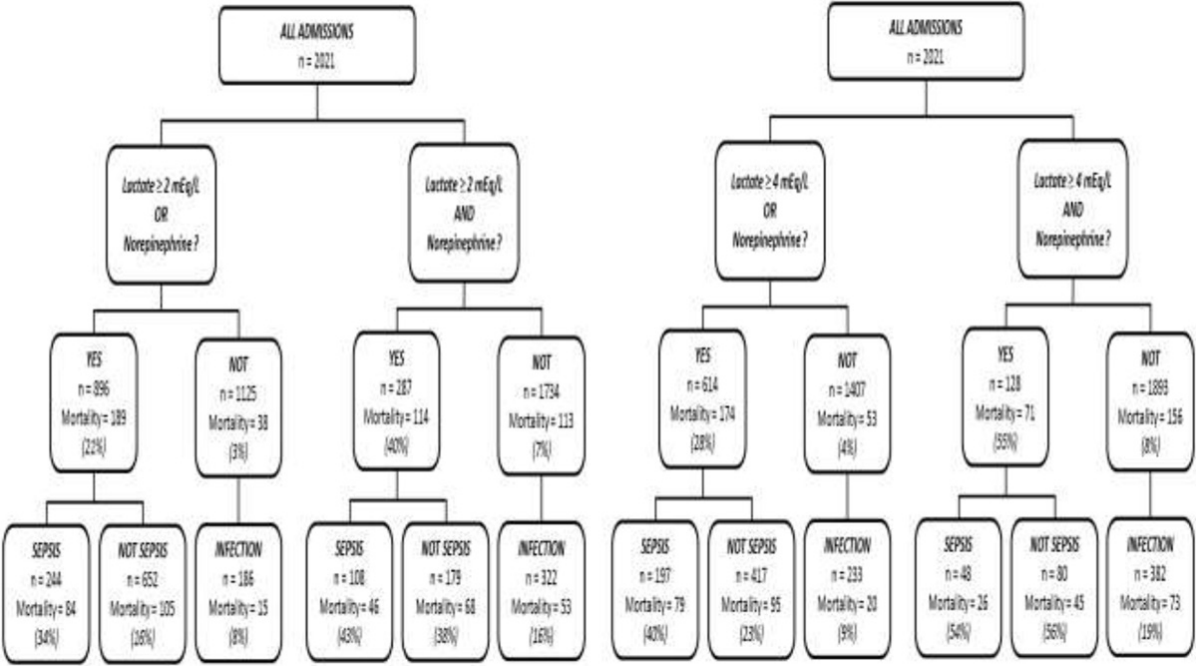
**Mortality rate across different groups**.

Of the 2021 patients included, 430 had a diagnosis of sepsis at admission; these patients had an ICU mortality of 23 %. One hundred and eighty-six needed norepinephrine during the first 24 hours and had a mortality rate of 40 %, 166 had an initial lactate ≥2 mEq/l with a mortality rate of 34 %, and 59 had an initial lactate ≥4 mEq/l with a mortality rate of 53 %. Using a lactate threshold of ≥2 mEq/l, patients in the group AND needed higher norepinephrine doses (first 24 hours) (0.39 (0.16-0.76) vs. 0.14 (0.03-0.44) μg/kg/minute), and ≥4 mEq/l yielded similar results. The mortality rates are shown in Figure 1. Patients in the group AND had higher mortality rates than the group OR, but there was a much smaller number of patients. Patients admitted with an infection but not fulfilling the criteria for the group AND or group OR had a lower mortality rate.

## Conclusion

Mortality in our septic population was higher than that reported in recent randomized controlled trials for early sepsis resuscitation in the ER [1,2], limiting the external validity of these trial results to other ICU populations. Mortality was higher when hyperlactatemia and need for norepinephrine were present simultaneously compared with the presence of only one of these two criteria.
